# Latent profile analysis and principal axis factoring of the DSM-5 dissociative subtype

**DOI:** 10.3402/ejpt.v6.26406

**Published:** 2015-04-01

**Authors:** Paul A. Frewen, Matthew F. D. Brown, Carolin Steuwe, Ruth A. Lanius

**Affiliations:** 1 Department of Psychiatry, Western University, London, Ontario, Canada; 2 Department of Psychology, Western University, London, Ontario, Canada; 3 Graduate Program in Neuroscience, Western University, London, Ontario, Canada; 4 Research Department, Clinic of Psychiatry, Ev. Krankenhaus Bielefeld, Bielefeld, Germany

**Keywords:** Posttraumatic stress disorder, dissociative subtype, dissociation, trauma-related altered states of consciousness, psychological trauma

## Abstract

**Objective:**

A dissociative subtype has been recognized based on the presence of experiences of depersonalization and derealization in relation to DSM-IV posttraumatic stress disorder (PTSD). However, the dissociative subtype has not been assessed in a community sample in relation to the revised DSM-5 PTSD criteria. Moreover, the 20-item PTSD Checklist for DSM-5 (PCL-5) currently does not assess depersonalization and derealization.

**Method:**

We therefore evaluated two items for assessing depersonalization and derealization in 557 participants recruited online who endorsed PTSD symptoms of at least moderate severity on the PCL-5.

**Results:**

A five-class solution identified two PTSD classes who endorsed dissociative experiences associated with either 1) severe or 2) moderate PTSD symptom severity (D-PTSD classes). Those in the severe dissociative class were particularly likely to endorse histories of childhood physical and sexual abuse. A principal axis factor analysis of the symptom list identified six latent variables: 1) Reexperiencing, 2) Emotional Numbing/Anhedonia, 3) Dissociation, 4) Negative Alterations in Cognition & Mood, 5) Avoidance, and 6) Hyperarousal.

**Conclusions:**

The present results further support the presence of a dissociative subtype within the DSM-5 criteria for PTSD.

The diagnostic criteria for posttraumatic stress disorder (PTSD) have undergone extensive revision within the fifth edition of the *Diagnostic and Statistical Manual of Mental Disorders* (DSM-5; American Psychiatric Association [APA], 2013). Not only are four clusters of PTSD symptoms now recognized (i.e., reexperiencing, avoidance, negative alterations in cognitions and mood, and hyperarousal; Friedman, 2013) but further a dissociative subtype of PTSD (D-PTSD) is included based on clinical, psychometric, and neurobiological research identifying it as a unique PTSD phenotype (e.g., Lanius, Brand, Vermetten, Frewen, & Spiegel, 2012; Lanius, Wolf, et al., 2014; Lanius et al., 2010; Stein et al., 2013; Wolf, Miller, et al., 2012). The dissociative subtype is diagnosed based on patient reports of depersonalization (i.e., a subjective state of feeling that one is disconnected or detached from his or her body [APA, 2013]) and derealization (i.e., a subjective state of perceiving the world as if it is not real, or is characteristically altered in some way, for example, seeming distorted, dreamlike, or foggy [APA, 2013]). Previous research has suggested prevalence rates for the dissociative subtype typically ranging between 14 and 35% in persons with PTSD (Armour, Karstoft, & Richardson, 2014; Blevins, Weathers, & Witte, 2014; Stein et al., 2013; Steuwe, Lanius, & Frewen, 2012; Wolf, Lunney, et al., 2012; Wolf, Miller, et al., 2012; Wolf et al., 2014) depending on the presence of certain risk factors including gender (e.g., often higher estimates have been found in women; Wolf, Lunney, et al., 2012; Steuwe et al., 2012 c.f., Stein et al., 2013) and type and severity of trauma exposure (e.g., higher estimates in persons exposed to more severe histories of childhood and sexual trauma; for example, Stein et al., 2013; Steuwe et al., 2012; Wolf, Miller, et al., 2012). However, latent profile analyses identifying D-PTSD have only been undertaken in relation to the PTSD criteria of DSM-IV, and studies of associations between depersonalization and derealization and the revised DSM-5 criteria have only been completed in college students to date (Armour, Contractor, Palmieri, & Elhai, 2014). Critically, whether a dissociative subtype of PTSD is evident in relation to the DSM-5 criteria within larger community samples requires investigation.

Previous studies of D-PTSD have assessed dissociation in various ways, for example, via items from the *Clinician-Administered PTSD Scale* (e.g., Steuwe et al., 2012; Wolf, Miller, et al., 2012), select items from the *Dissociative Experiences Scale* (Stein et al., 2013), and other self-report measures of dissociative experiences (e.g., subscales from the *Multiscale Dissociation Inventory* (MDI; Blevins et al., 2014) and trauma symptoms (e.g., the *Trauma Symptom Inventory*; Wolf, Lunney, et al., 2012). Variability in operationalization of the dissociative subtype across studies can contribute to heterogeneity in findings, rendering a cumulative interpretation of results more difficult. Moreover, clinicians seeking to assess the dissociative subtype by self-report currently lack a practical, brief method by which to do so. Acknowledging that previous versions of the *PTSD Symptom Checklist* (PCL) have historically been among the most often used self-report measures of PTSD symptoms in both research and practice (e.g., Elhai, Gray, Kashdan, & Franklin, 2005), it seems likely that the recently developed *PTSD Symptom Checklist for DSM-5* (PCL-5; Hoge, Riviere, Wilk, Herrell, & Weathers, 2014; Weathers et al., 2013) will be an often-used measure of self-reported PTSD symptomatology under the revised DSM-5 criteria. However, a limitation of the PCL-5 is that it currently does not include items appropriate to the assessment of the dissociative subtype of PTSD (i.e., it does not include items measuring depersonalization or derealization).

The proposed structure of PTSD symptoms within the DSM-5, although largely consistent with extant literature on the factor structure of DSM-IV PTSD symptoms (see Friedman, 2013), will undoubtedly lead to changes in estimates of latent structure. To date, the few studies examining the factor structure of PTSD have largely supported the DSM-5 factor structure with differences generally attributable to the factor of “Negative Alterations in Cognition and Mood,” and the poor fit of “reckless behavior,” “psychogenic amnesia,” and “flashbacks” items (Biehn et al., 2013; Contractor et al., 2014; Elhai et al., 2012; Gentes et al., 2014; Liu et al., 2014; Miller et al., 2013). There is further a long-standing debate regarding the relationship between dissociation and the core features of PTSD. Steuwe, Lanius, and Frewen's (2012) results support considering experiences of depersonalization and derealization as an independent latent variable which is moderately intercorrelated with the core PTSD factors of DSM-IV. It is therefore of further interest to investigate whether depersonalization and derealization represent a unique factor that is intercorrelated with the core PTSD symptoms of DSM-5 as well, and whether differences between latent classes within persons with PTSD can be attributed to differences on latent PTSD symptom factors.

To support measurement of dissociative experiences in persons assessed for DSM-5 PTSD as standard practice, and to investigate the relationship between DSM-5 PTSD symptomatology and dissociative experiences including depersonalization and derealization, we therefore evaluated a two item list that could be used to supplement the PCL-5. Specifically, the current research evaluated whether a dissociative subtype could be identified in relation to the DSM-5 PTSD criteria in a latent profile analysis of the PCL-5 measuring not only the 20 core PTSD criteria but also inclusive of two additional items measuring depersonalization and derealization. We hypothesized that persons experiencing depersonalization and/or derealization would demark a distinct D-PTSD latent class as has been found in previous studies. Moreover, we conducted a principal axis factor analysis and hypothesized to identify a latent variable for dissociative experiences of depersonalization and/or derealization as well as factors distinguishing between PTSD symptoms of reexperiencing, avoidance, negative alterations in cognitions and mood, and hyperarousal, in concordance with the DSM-5 model.^1^

## Methods

### Participants

A total of 2,728 participants intending to represent a general population sample were recruited across three waves of data collection (*n*_Wave__1_=1,115; *n*_Wave__2_=705; *n*_Wave__3_=908) using Amazon's Mechanical Turk (MTurk) web service which has been validated as a recruitment strategy for mental health research (Shapiro, Chandler, & Mueller, 2013). Individuals freely volunteered to participate in our study after reading a brief advertisement of the study posted alongside other studies. Participants received a nominal compensation for the time required to complete the study via registration of their unique MTurk username.

Participants were first asked to complete demographic information as well as the PCL-5, followed by additional measures for the purposes of further hypothesis testing and sample characterization, the latter varying across three waves of data collection. A total of 2,507 participants (91.9%) completed demographics and the PCL-5, with 2,136 (85.2%) of these participants also completing the additional study measures specific to each of the three study waves (see Procedure section). Participants who completed the full study battery did not differ from non-completers on any demographic measure or in terms of overall PCL-5 PTSD symptom severity. The present study, however, examines only those 557 participants (i.e., 22.2% of the total sample; *n*_Wave__1_=243; *n*_Wave__2_=131; *n*_Wave__3_=183) who scored at or above the recently recommended cut-off score of 38 for probable PTSD diagnosis on the PCL-5 (Hoge et al., 2014; Weathers et al., 2013); data referring to the full sample will be presented elsewhere.

The final sample (*n*=557) consisted of mainly female participants (*n*=395; 70.9%) versus male participants (*n*=159; 28.5%), with three participants (0.5%) declining to describe their sex. Participants were generally of middle age (*M*=33.10, *SD=*10.80), and Caucasian (*n=*412; 73.9%), Mixed (*n*=54; 9.7%), or one of a number of specific ethnicities (*n*=71; 12.9%); nine participants failed to indicate their ethnic background (1.6%). A majority of participants were married (*n*=146; 26.2%) or single (*n*=275; 49.4%), with 113 (23.1%) endorsing being either “separated” (*n*=19), “divorced” (*n*=61), “widowed” (*n*=4), “common-law” (*n*=29), or “other” (*n*=15); 19 participants did not report on their marital status (3.4%). Most participants were currently employed part-time or full time (*n*=260; 46.7%), were self-employed (*n*=78; 14.0%), or listed “student” as their current primary working role (*n*=72; 12.9%). By contrast, 90 participants endorsed currently being unemployed (16.2%), and 42 described themselves as unable to work (7.5%); nine participants endorsed an employment status of “other” (1.6%), or declined to respond (*n*=4; 0.7%). A majority of participants had partially completed post-secondary education (*n*=484; 86.9%), with a minority completing secondary school (*n*=59; 10.6%), not completing high school (*n*=9; 1.6%), or declining to respond (*n*=5; 0.9%). A total of 63% of participants (*n=*348) reported suffering from a diagnosed psychological problem either currently (*n=*248, 44.5%) or sometime in the past but not currently (*n*=100, 18%); the remaining participants either denied any history of diagnosed psychological disorders (*n=*185, 33.2%) or declined to comment (*n=*24, 4.3%).

### Measures

#### PTSD Checklist for DSM-5 (PCL-5)

The 20-item PCL-5 (Weathers et al., 2013) was administered to participants from all study waves to measure each of the DSM-5 PTSD symptoms. Responses were made on a past month frequency scale from 0 (*Not at all*) to 4 (*Extremely*). Scores on the total PCL-5 therefore range from 0 to 80, with higher scores indicating greater severity of PTSD symptoms and a score of 38 recommended as a cut-off for probable PTSD, corresponding to a PCL-S (DSM-IV) score of 50 (Hoge et al., 2014; Weathers et al., 2013). The reliability of the total PCL-5 in the current sample was adequate (*α*=0.76).

#### Dissociation-TRASC item list

Using the same item anchors and instruction line as for the PCL-5, we appended 10 items to the 20-item PCL-5 (i.e., as items 21–30) to measure various trauma-related dissociative experiences, two of which were intended specifically as measures of depersonalization and derealization indicative of the DSM-5 PTSD dissociative subtype and are examined herein. These items were phrased as follows: “Out of Body Experience—Feeling detached or separated from your body, for example, feeling like you are looking down on yourself from above, or like you are an outside observer of your own body” (i.e., measuring depersonalization) and “Feeling like what you are experiencing is not real—A change in the way you perceive or experience the world or other people, so that things seem dreamlike, strange, or unreal” (i.e., measuring derealization). An additional eight items were also administered to assess the broader domain of dissociative experiences, including what have been recently termed *trauma-related altered states of consciousness* (TRASC; Frewen & Lanius, 2014, 2015); results pertaining to these eight additional items, however, will be described elsewhere. The phrasing of the 10 items was developed rigorously based on feedback from clinicians and researchers with expertise in PTSD and dissociative disorders, as well as from patients attending a psychological trauma clinical research service with which the first and last authors are affiliated.

### Additional measures of dissociative experiences

#### Cambridge Depersonalization Scale (CDS)

The CDS (Sierra & Berrios, 2000) is a 29-item self-report measure of the frequency of depersonalization experiences over the past 6 months. Responses are made on a scale from 0 (*Never*) to 4 (*All the time*) with higher scores representing a greater frequency of depersonalization experiences. Factor analyses of the CDS have demonstrated that the scale is multidimensional with studies reporting on two (Blevins, Witte, & Weathers, 2013), four (Apontze-Soto, Vélez-Pastrana, Martínez-Taboas, & González, 2014; Sierra, Baker, Medford, & David, 2005), and five factors (Simeon, Smith, Knutelska, & Smith, 2008). Due to the replication and conciseness of the original four factor solution, the current study reports subscale scores for *Anomalous Body Experience* (nine items; e.g., “Seeing oneself outside, as if looking in a mirror”; *α*=0.94), *Emotional Numbing* (six items; e.g., “No emotions felt when weeping or laughing”; *α*=0.87), *Alienation from Surroundings* (four items; e.g., “Surroundings feel detached or unreal”; *α*=0.86), and *Anomalous Subjective Recall* (five items; e.g., “Personal memories feel as though one has not been involved in them”; *α*=0.85).

#### Multiscale Dissociation Inventory (MDI)

The MDI (Briere, 2002) is a 30-item self-report instrument designed to measure a diverse set of dissociative experiences. The MDI is broken down into six five-item subscales: *Depersonalization* (e.g., “Your body feeling like it was someone else's”; *α*=0.91), *Derealization* (e.g., “Things around you suddenly seeming not quite right, a little bit off”; *α*=0.92), *Emotional Constriction* (e.g., “Not having any emotions or feelings at a time when you should have been upset”; *α*=0.92), *Disengagement* (e.g., “Driving or walking without noticing where you were going”; *α*=0.85), *Memory Disturbance* (e.g., “Suddenly realizing that hours had gone by and not knowing what you had been doing during that time”; *α*=0.87), and *Identity Dissociation* (e.g., “Feeling like there was more than one person inside of you”; *α*=0.94). Responses are made on a 5-point Likert scale ranging from 1 (*Never*) to 5 (*Very Often*), with higher scores indicating a greater frequency of a given dissociative experience over the past month.

#### Multidimensional Inventory of Dissociation (MID)

The full MID (Dell, 2006; Dell & Lawson, 2009) is a 218-item self-report, multiscale measure of pathological dissociation. Responses are made on an 11-point scale ranging from 0 (*Never*) to 10 (*Always*). Importantly, a time frame for the frequency of these experiences is not specified, similar to the *Dissociative Experiences Scale*. Administration of the full MID was considered too lengthy for the present study. For this study, we examined responses only to a subset of subscales from the MID which measured depersonalization and derealization as well as the conceptually related phenomena of trance experiences and time loss. Toward these goals, the following subscales were administered: *Depersonalization* (12 items, e.g., “Standing outside your body watching yourself, as if you were another person”, *α*=0.95), *Derealization* (12 items; e.g., “Being in a familiar place, but finding it strange or unfamiliar”, *α*=0.94), *Trance* (12 items; e.g., “Having trance-like episodes where you stare off into space and lose awareness for what is going on around you”, *α*=0.93), and *Time Loss* (4 items; e.g., “Having blank spells or black outs in your memory”, *α*=0.86). In addition, the MID *Emotional Suffering* subscale (12 items; e.g., “Feeling empty and painfully alone”, *α*=0.91) was administered as a presumed measure of *non*-dissociative distress, described further below.

### Measures of presumed non-dissociative distress

#### Difficulty in Emotion Regulation Scale (DERS)

The DERS (Gratz & Roemer, 2004) is a 36-item self-report scale designed to measure difficulties in emotion regulation. Responses are made on a scale from 0 (*almost never* [*0–10%*]) to 5 (*almost always* [*91–100%*]). Factor analyses differentiate between items reflecting difficulties in: 1) awareness and understanding of emotions; 2) the ability to engage in goal-directed behavior in the context of emotional distress; 3) acceptance of emotions; 4) refraining from impulsive behavior when experiencing negative emotions; and 5) access to emotion regulation strategies. However, to simplify presentation and reduce the number of statistical tests, only the total DERS score (*α*=0.96) was analyzed in this study.

#### Inventory of Interpersonal Problems (IIP-32)

The IIP (Barkham, Hardy, & Startup, 1996) is a 32-item measure designed to be a short form to the full 127-item IIP (Barkham, Hardy, & Startup, 1994). The IIP measures difficulties individuals’ experience in their interpersonal relationships. Responses range from 0 (*Not at all*) to 5 (*Extremely*) indicating how much the respondent has had trouble with the given item over the course of their life. For the purposes of this study, we only examined IIP total scores (*α*=0.93).

#### MID emotional suffering subscale

Whereas the majority of MID items are intended as measures of pathological dissociation (Dell, 2006; Dell & Lawson, 2009), this instrument also includes 12 items intended as a measure of general *Emotional Suffering*, which for the purposes of the present study were therefore presumed as an indicator of non-specific, non-dissociative distress. Example items from the MID *Emotional Suffering* subscale include: “Feeling empty and painfully alone,” “Feeling mad,” and “Feeling hurt.” The internal consistency in the present study was *α*=0.91.

### Measures of childhood trauma history

#### Childhood Trauma Questionnaire (CTQ)

The CTQ (Bernstein et al., 2003) is a 28-item self-report instrument that measures experiences of *Emotional Abuse* (*α*=0.91), *Physical Abuse* (*α*=0.85), *Sexual Abuse* (*α*=0.96), as well as experiences of *Emotional Neglect* (*α*=0.91) and *Physical Neglect* (*α*=0.79). Responses are made on a 5-point Likert scale ranging from 0 to 5 (*Never True* to *Very Often True*), indicating severity of experiences.

#### Childhood Trauma Questionnaire-Screen (CTQ-S)

This included only four items from the CTQ, two of which were previously validated (Thombs, Bernstein, Ziegelstein, Bennett, & Walker, 2007) for screening history of physical abuse (i.e., “People in my family hit me so hard that it left me with bruises or marks”) and sexual abuse (i.e., “Someone tried to touch me in a sexual way, or tried to make me touch them”). Following Frewen et al. (2013), we also used a face valid screening item for emotional abuse history (i.e., “I believe that I was emotionally abused”) and presented but did not analyze a filler item assessing general satisfaction with familial upbringing (“i.e., My family was a source of strength and support”) (Frewen et al., 2013). Responses were made on the same 5-point scale as used for the CTQ as described previously.

#### Juvenile Victimization Questionnaire (JVQ) adult retrospective version

The JVQ (Hamby, Finkelhor, Ormrod, & Turner, 2004) is a 34-item measure designed to assess a broad range of childhood traumatic experiences including not only childhood maltreatment but also experiences of criminal victimization (e.g., robbery), sexual assault, bullying, and witnessing violence. Responses to the JVQ items are based on frequency and/or severity of victimization experiences, with responses ranging on a six-point Likert scale anchored from 0 (*No*) to 5 (*5 times or more*). The JVQ often is delineated into five subscales (i.e., *Conventional Crime* [*α*=0.86], *Child Maltreatment* [*α*=0.69], *Peer and Sibling Victimization* [*α*=0.80], *Sexual Victimization* [*α*=0.84], and *Witnessing Violence* [*α*=0.82]), which have demonstrated reliability in previous research (Finkelhor, Hamby, Ormrod, & Turner, 2005).

### Procedure

The study procedure received approval by an academic research ethics board. All data collection occurred on a secure, encrypted website independent of the MTurk website to preserve participant anonymity and confidentiality. All participants completed demographic questions first, followed by the PCL-5, and the Dissociation-TRASC 10-item list, which included the aforementioned two items measuring depersonalization and derealization. Depending on the three waves of data collection in which participants took part, they then completed one of three additional measure of dissociative experiences (Wave 1: CDS, Wave 2: MDI, Wave 3: MID), one of three measures of presumed non-dissociative distress (Wave 1: DERS, Wave 2: IIP, Wave 3: MID-Emotional Suffering), and one of three measures of childhood trauma history (Wave 1: CTQ, Wave 2: JVQ, Wave 3: CTQ-S). It should be noted that all participants also completed the *Childhood Attachment and Relational Trauma Screen* (CARTS; Frewen et al., 2013), although limitations in manuscript length necessitate that results pertaining to the CARTS be presented elsewhere.

### Statistical analysis

We first conducted an exploratory principal axis factor analysis (EFA) on the 22 items (i.e., 20 core PCL-5 items plus the appended depersonalization and derealization items) and interpreted following a direct oblimin rotated solution allowing for factors to correlate (Fabrigar et al., 1999) as was expected from prior research with DSM-IV PTSD symptoms (Steuwe et al., 2012). An EFA was preferred over a confirmatory factor analysis given that the factor structure of the 20 core symptoms of DSM-5 PTSD remains to be extensively validated to date. Predicted factor scores were calculated from the obtained factor loadings via multiple regression and compared among the latent classes.

Then, we conducted latent profile analysis (LPAs) on the 20 core DSM-5 PTSD criteria (measured by the standard PCL-5) in addition to the two depersonalization and derealization items from our 10-item Dissociation-TRASC supplement. We replicated procedures for LPA as implemented by Steuwe et al. (2012) via MPlus, Version 5.0 (Muthén & Muthén, 2007). We estimated LPAs increasing from two classes via the maximum likelihood method with robust standard errors and compared the loglikelihood and entropy values obtained in addition to indices of model fit with specific preference to the Bayesian information criterion (BIC; Nylund, Asparouhov, & Muthén, 2007; Schwarz, 1978) and the bootstrap likelihood ratio test (BLRT; McLachlan & Peel, 2000); the Lo-Mendell–Rubin adjusted likelihood ratio test (LMR-A; Lo, Mendell, & Rubin, 2001) was also performed (for rationale see Steuwe et al., 2012). Participants were assigned to their most likely class in accordance with the model accepted and compared concerning PTSD severity, dissociative symptoms measured by other instruments, and trauma history via ANOVA; all post-hoc-analyses were corrected for multiple comparisons (Bonferroni). Should the LPA identify a dissociative subtype as hypothesized, to facilitate ease of scoring and application, we also aimed to determine the PCL-5 Likert-scale scores for the depersonalization and derealization items that achieved the greatest balance between sensitivity and specificity for the dissociative class as per the DSM-5 algorithm (i.e., requiring the endorsement of *either* depersonalization or derealization).

## Results

### Principal axis factoring

Principal axis factoring with oblimin rotation identified six latent variables using the eigenvalue greater than one criterion, rendering a solution that collectively explained 41.97% of the variance (see Table 1). Reference to the pattern and structural matrices obtained supports the following interpretation: the first factor obtained high loadings on only the reexperiencing symptoms of PTSD (PCL-5 items 1–5) and was therefore labeled *Reexperiencing* (explaining 16.53% of the variance); the second factor obtained high loadings on only the emotional numbing or anhedonia symptoms of PTSD (PCL-5 items 12–15) and was therefore labeled *Emotional numbing/Anhedonia* (explaining 8.10% of the variance); the third factor obtained high loadings on only the depersonalization and derealization symptoms and was therefore labelled *Dissociation* (explaining 6.59% of the variance); the fourth factor obtained high loadings on only the negative cognition and pervasive negative emotional states of PTSD (PCL-5 items 9–11) and can therefore be labeled *Negative Alterations in Cognition and Mood* (explaining 3.88% of the variance); the fifth factor obtained high loadings on the avoidance symptoms of PTSD (PCL-5 items 6–7) and can therefore be labeled *Avoidance* (explaining 3.77% of the variance); and finally, the sixth factor obtained high negative loadings—only on the hypervigilance and startle reactivity symptoms of PTSD (PCL-5 items 17–18)—and can therefore be labeled as a specific *Hyperarousal* factor similar to that identified by Simms and colleagues’ for DSM-IV PTSD (Simms, Watson, & Doebbelling, 2002; Yufik & Simms, 2010) (explaining 3.35% of the variance). Table 2 reports the factor correlation matrix obtained; interestingly, factor correlations were generally small or non-significant, ranging from −0.31 to 0.31 (*M*=0.02, *SD=*0.20). Predicted scores on the six factors were generated from the factor loadings using multiple regression for use in comparison between classes identified by LPA (see below).

**Table 1:** Principal axis factor analysis with direct oblimin rotation of the 20-item PCL-5 plus depersonalization and derealization items

	Communalities		Factor Matrix						Pattern Matrix						Structure Matrix					
			
Items/variables	Init	Extr	1	2	3	4	5	6	1	2	3	4	5	6	1	2	3	4	5	6
1. Intrusive memories	0.41	0.50	0.53	−0.19	−0.28	−0.19	−0.26	0.01	0.69	0.04	0.00	0.05	0.07	0.12	0.69	0.12	0.16	0.15	0.27	−0.13
2. Nightmares	0.32	0.33	0.47	−0.28	−0.03	−0.18	−0.06	0.03	0.45	0.00	0.14	−0.14	0.14	−0.03	0.53	0.08	0.28	−0.10	0.28	−0.24
3. Flashbacks	0.35	0.45	0.50	−0.26	−0.09	−0.13	−0.32	−0.10	0.66	−0.03	0.12	−0.01	−0.11	0.00	0.65	0.08	0.28	0.05	0.10	−0.21
4. Upset at reminder	0.41	0.51	0.52	−0.16	−0.38	−0.15	−0.18	−0.14	0.68	0.03	−0.16	0.11	0.06	−0.04	0.69	0.12	0.03	0.21	0.28	−0.22
5. Phys. reaction at reminder	0.36	0.41	0.50	−0.24	−0.19	0.00	−0.06	−0.14	0.50	−0.10	0.01	0.10	0.07	−0.22	0.60	0.10	0.20	0.16	0.29	−0.37
6. Av. Int. R.	0.34	0.49	0.38	−0.16	−0.27	−0.05	0.37	0.32	0.05	0.01	0.05	0.02	0.68	0.01	0.27	0.05	0.11	0.10	0.69	−0.17
7. Av. Ext. R.	0.38	0.57	0.46	−0.17	−0.26	−0.06	0.39	0.33	0.70	0.03	0.09	0.01	0.71	−0.01	0.32	0.09	0.16	0.11	0.74	−0.22
8. Amnesia	0.19	0.20	0.29	−0.14	0.20	0.13	0.04	0.20	−0.02	−0.06	0.42	0.03	0.16	−0.02	0.14	0.03	0.42	0.03	0.19	−0.17
9. Negative beliefs	0.33	0.53	0.31	0.37	−0.19	0.48	−0.15	0.07	−0.04	0.08	0.15	0.71	−0.03	−0.01	0.09	0.21	0.13	0.71	0.05	−0.06
10. Blaming	0.30	0.41	0.28	0.22	−0.36	0.37	−0.13	0.01	0.12	−0.03	−0.03	0.61	0.02	−0.01	0.19	0.08	−0.02	0.63	0.13	−0.04
11. Neg. emotions	0.34	0.39	0.37	0.35	−0.28	0.22	0.04	−0.05	0.05	0.20	−0.10	0.50	0.09	−0.14	0.19	0.31	−0.02	0.55	0.20	−0.20
12. Loss of interest	0.38	0.53	0.32	0.57	0.05	−0.30	0.08	0.03	−0.03	0.74	−0.07	0.01	0.09	0.05	0.07	0.72	0.04	0.13	0.10	−0.12
13. F. distant/cut−off	0.42	0.55	0.29	0.67	0.00	−0.14	0.03	0.05	−0.10	0.70	−0.05	0.21	0.03	0.07	0.00	0.70	0.02	0.31	0.05	−0.06
14. Anhedonia	0.32	0.39	0.34	0.44	0.13	−0.20	−0.14	0.05	0.10	0.59	0.11	0.07	−0.08	0.12	0.16	0.60	0.20	0.16	−0.03	−0.07
15. Irritable/Anger	0.22	0.25	0.34	0.28	0.21	−0.09	−0.00	−0.09	0.02	0.40	0.12	0.03	−0.09	−0.14	0.13	0.46	0.23	0.08	−0.02	−0.26
16. Risk taking	0.24	0.26	0.29	0.00	0.39	−0.04	−0.14	−0.01	0.09	0.16	0.37	−0.09	−0.17	−0.07	0.16	0.23	0.44	−0.09	−0.11	−0.21
17. Hypervigilance	0.31	0.43	0.42	−0.13	0.12	0.19	0.28	−0.34	0.02	−0.10	0.05	0.08	0.02	−0.65	0.23	0.08	0.23	0.07	0.17	−0.64
18. Startle reactivity	0.36	0.59	0.50	−0.09	0.21	0.14	0.30	−0.42	0.02	−0.00	0.05	0.04	−0.03	−0.75	0.26	0.19	0.29	0.04	0.15	−0.76
19. Diff. concentrating	0.21	0.21	0.31	0.16	0.19	−0.12	0.18	−0.11	−0.04	0.31	0.06	−0.08	0.05	−0.25	0.10	0.36	0.18	−0.03	0.10	−0.34
20. Insomnia	0.12	0.10	0.23	0.03	0.03	−0.15	0.11	−0.07	0.09	0.18	−0.03	−0.10	0.08	−0.14	0.16	0.21	0.07	−0.05	0.14	−0.22
Depersonalization	0.49	0.71	0.50	−0.21	0.56	0.17	−0.17	0.22	0.07	−0.03	0.82	−0.01	−0.06	−0.03	0.26	0.11	0.83	0.28	0.04	0.01
Derealization	0.42	0.49	0.50	−0.07	0.38	0.16	−0.05	0.25	0.01	0.07	0.66	0.08	0.09	−0.02	0.22	0.18	0.68	0.26	−0.07	0.15

Factors were interpreted as follows: 1) Reexperiencing, 2) Emotional Numbing/Anhedonia, 3) Dissociation, 4) Negative Alterations in Cognition & Mood, 5) Avoidance, and 6) Hyperarousal.

**Table 2:** Six factor correlation matrix identified in principal axis factor analysis of the 20-item PCL-5 plus depersonalization and derealization items

	1	2	3	4	5	6
1) Reexperiencing	1.00					
2) Emotional numbing/anhedonia	0.14	1.00				
3) Dissociation	0.26	0.17	1.00			
4) Negative alterations in cognition & mood	0.12	0.16	−0.03	1.00		
5) Avoidance	0.31	0.05	0.07	0.12	1.00	
6) Hyperarousal	−0.31	−0.24	−0.31	−0.01	−0.22	1.00

### Latent profile analysis

The LPA, conducted on the 20 PCL-5 items assessing the core symptoms of DSM-5 PTSD, as well as the derealization and depersonalization items, suggested improving model fit for solutions of up to five classes; a model with six classes was rejected given that the best loglikelihood value was not replicated (i.e., versus the five-class model; see Table 3). Relative to models of four or fewer classes, the best fitting model was the five-class solution, which exhibited the lowest loglikelihood value=−17102.39, the lowest BIC=36963.21, the highest entropy=0.872, and an improved BLRT<0.001, relative to the four-class solution.

**Table 3:** Fit indices for different latent class solutions (PCL-5 ≥38)

Model	Loglikelihood	BIC	Entropy	LMR-A *p*-value	BLRT *p*-value
Two classes	−18561.07	37545.76	0.849	<0.001	<0.001
Three classes	−18326.61	37222.25	0.826	0.002	<0.001
Four classes	−18202.34	37119.14	0.816	0.475	<0.001^a^
Five classes	−18051.67	36963.21	0.872	0.041	<0.001
Six classes	−17961.87^a^	36929.03	0.886	0.469	<0.001

BIC=Bayesian information criterion; LMR-A=Lo-Mendell-Rubin adjusted likelihood ratio test; BLRT=bootstrap likelihood ratio test.

a The best loglikelihood value was not replicated. As we used a high number of starts, this indicates that too many classes were extracted with a six-class solution.

**Fig. 1:**
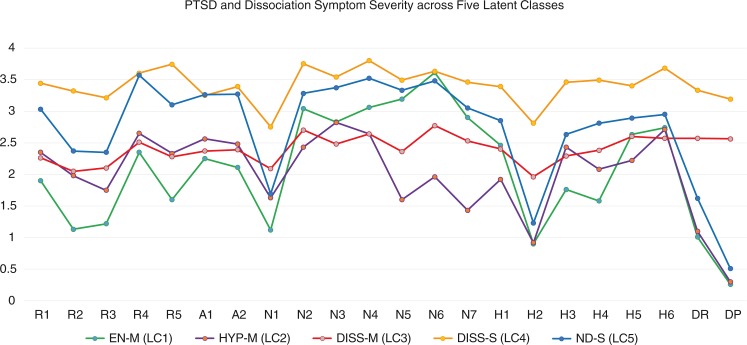
PTSD and dissociation symptom severity across five latent classes. EN-M=Emotional Numbing—Moderate. HYP-M=Hyperarousal—Moderate. DISS-M=Dissociation—Moderate. DISS-S=Dissociation—Severe. ND-S=Non-Dissociative—Severe. LC=Latent Class. Statistically significant between class differences are reported in Table 4.

The resulting five classes were interpreted in reference to the measures with which they were extracted (Asparouhov & Muthen, 2013, e.g., Wolf, Miller, et al., 2012), and a multivariate ANOVA was significant as such, *F*(88, 2,136)=25.41, *p<*0.001, *η* ^2^−partial=0.51. Follow-up univariate ANOVAs identified significant differences (*p*<0.001) between the five classes for all 20 of the core DSM-5 PTSD symptoms as well as for experiences of depersonalization and derealization. Table 4 presents the results of post-hoc tests. Please see Fig. 1 for an illustration of the five PTSD symptom profiles.

**Table 4:** Differences between latent classes in PTSD and depersonalization and derealization symptom frequency

	a. Emotional numbing	b. Hyperarousal	c. Moderate D-PTSD	d. Severe D-PTSD	e. Severe ND-PTSD
1. Intrusive memories	1.90 (1.00)^b^,^c^,^d^,^e^	2.35 (0.84)^a^,^d^,^e^	2.26 (0.96)^a^,^d^,^e^	3.43 (0.76)^a^,^b^,^c^,^e^	3.03 (0.81)^a^,^b^,^c^,^d^
2. Nightmares	1.13 (1.11)^b^,^c^,^d^,^e^	1.98 (1.23)^a^,^d^	2.05 (1.04)^a^,^d^	3.32 (0.71)^a^,^b^,^c^,^e^	2.37 (1.33)^a^,^d^
3. Flashbacks	1.22 (1.06)^b^,^c^,^d^,^e^	1.75 (1.09)^a^,^d^,^e^	2.10 (0.99)^a^,^d^	3.21 (0.84)^a^,^b^,^c^,^e^	2.34 (1.17)^a^,^b^,^d^
4. Upset at reminder	2.35 (0.94)^b^,^d^,^e^	2.65 (0.87)^a^,^d^,^e^	2.51 (0.90)^d^,^e^	3.60 (0.59)^a^,^b^,^c^	3.57 (0.65)^a^,^b^,^d^
5. Phys. React. at Reminder	1.60 (0.93)^b^,^c^,^d^,^e^	2.33 (1.05)^a^,^d^,^e^	2.28 (0.93)^a^,^d^,^e^	3.74 (0.52)^a^,^b^,^c^,^e^	3.10 (0.91)^a^,^b^,^c^,^d^
6. Avoid. Int. Reminders	2.25 (1.07)^d^,^e^	2.56 (0.95)^d^,^e^	2.37 (0.96)^d^,^e^	3.25 (0.99)^a^,^b^,^c^	3.26 (0.83)^a^,^b^,^c^
7. Avoid. Ext. Reminders	2.11 (1.14)^b^,^d^,^e^	2.48 (1.00)^a^,^d^,^e^	2.39 (0.95)^d^,^e^	3.39 (0.84)^a^,^b^,^c^	3.27 (0.87)^a^,^b^,^c^
8. Amnesia	1.12 (1.24)^b^,^c^,^d^,^e^	1.63 (1.21)^a^,^d^	2.09 (1.25)^a^,^d^	2.75 (1.46)^a^,^b^,^c^,^e^	1.69 (1.49)^a^,^d^
9. Negative beliefs	3.04 (1.14)^b^,^d^	2.43 (1.04)^a^,^d^,^e^	2.70 (0.99)^d^,^e^	3.75 (0.47)^a^,^b^,^c^,^e^	3.28 (1.11)^b^,^c^,^d^
10. Blaming	2.83 (1.19)^d^,^e^	2.82 (0.92)^d^,^e^	2.48 (1.08)^d^,^e^	3.54 (0.76)^a^,^b^,^c^,^e^	3.37 (1.00)^a^,^b^,^c^
11. Negative emotions	3.06 (0.84)^b^,^c^,^d^,^e^	2.64 (0.94)^a^,^d^,^e^	2.64 (0.89)^a^,^d^,^e^	3.81 (0.40)^a^,^b^,^c^	3.52 (0.77)^a^,^b^,^c^
12. Loss of interest	3.19 (0.82)^b^,^c^	1.60 (0.95)^a^,^c^,^d^,^e^	2.36 (0.97)^a^,^b^,^d^,^e^	3.49 (0.66)^b^,^c^	3.33 (0.78)^b^,^c^
13. Feeling distant/cut-off	3.61 (0.57)^b^,^c^	1.96 (0.90)^a^,^c^,^d^,^e^	2.76 (0.88)^a^,^b^,^d^,^e^	3.63 (0.62)^b^,^c^	3.48 (0.72)^b^,^c^
14. Anhedonia	2.90 (1.03)^b^,^c^,^d^	1.42 (0.87)^a^,^c^,^d^,^e^	2.53 (0.94)^a^,^b^,^d^,^e^	3.46 (0.73)^a^,^b^,^c^	3.04 (1.13)^b^,^c^
15. Irritability/anger	2.46 (1.19)^b^,^d^,^e^	1.92 (1.02)^a^,^c^,^d^,^e^	2.40 (1.00)^b^,^d^,^e^	3.39 (0.96)^a^,^b^,^c^,^e^	2.85 (1.16)^a^
16. Risk taking	0.90 (1.13)^c^,^d^	0.92 (1.06)^c^,^d^	1.96 (1.19)^a^,^b^,^d^,^e^	2.81 (1.37)^a^,^b^,^c^,^e^	1.23 (1.36)^c^,^d^
17. Hypervigilance	1.76 (1.22)^b^,^c^,^d^,^e^	2.43 (1.15)^a^,^d^	2.29 (1.07)^a^,^d^	3.46 (0.76)^a^,^b^,^c^,^e^	2.63 (1.16)^a^,^d^
18. Startle reactivity	1.58 (1.20)^b^,^c^,^d^,^e^	2.08 (1.01)^a^,^d^,^e^	2.38 (1.06)^a^,^d^,^e^	3.49 (0.73)^a^,^b^,^c^,^e^	2.81 (1.13)^a^,^b^,^c^,^d^
19. Difficulty concentrating	2.63 (1.08)^b^,^d^	2.22 (1.10)^a^,^d^,^e^	2.60 (0.99)^d^	3.40 (0.75)^a^,^b^,^c^,^e^	2.89 (1.16)^b^,^d^
20. Insomnia	2.74 (1.32)^d^	2.71 (1.21)^c^	2.57 (1.12)^d^	3.68 (1.21)^a^,^b^,^c^,^e^	2.94 (1.21)^d^
Depersonalization	0.26 (0.52)^c^,^d^,^e^	0.30 (0.54)^c^,^d^	2.56 (0.83)^a^,^b^,^d^,^e^	3.19 (0.77)^a^,^b^,^c^,^e^	0.51 (0.68)^a^,^c^,^d^
Derealization	1.01 (1.11)^c^,^d^,^e^	1.01 (0.98)^c^,^d^,^e^	2.57 (0.96)^a^,^b^,^d^,^e^	3.33 (0.93)^a^,^b^,^c^,^e^	1.62 (1.33)^a^,^b^,^c^,^d^

D=Dissociative, ND=Non-Dissociative.

a Statistically significantly different from EN-M class

b Statistically significantly different from HYP-M class

c Statistically significantly different from DISS-M class

d Statistically significantly different from DISS-S class

e Statistically significantly different from ND-S class

In brief, class 1 (*n=*140, 25.1%) exhibited lower reexperiencing symptoms (specifically, nightmares, flashbacks, and physiological reactivity), low hyperarousal symptoms (particularly hypervigilance and startle reactivity), and low dissociative symptoms, but a higher severity of emotional numbing and anhedonia symptoms (PCL-5 items 11–14); this class was therefore termed an “Emotional Numbing—Moderate” (EN-M) class. Class 2 (*n=*106, 19.0%) exhibited moderate PTSD symptom severity overall except for evidencing a low severity of emotional numbing and anhedonia symptoms (PCL-5 items 12–15); this class was therefore termed a “Hyperarousal—Moderate” (HYP-M) class. Class 3 (*n=*126, 22.6%) reported an overall moderate level of PTSD symptom severity but endorsed the presence of dissociative experiences of depersonalization and derealization at a moderate intensity; this class was therefore termed “Dissociative—Moderate” (DISS-M). Class 4 (*n=*57, 10.2%) not only reported the overall highest PTSD symptom severity but also endorsed frequent dissociative experiences of depersonalization and derealization; this class was therefore termed “Dissociative—Severe” (DISS-S). Finally, class 5 (*n=*128, 23.0%) exhibited an overall high PTSD symptom severity but did not endorse dissociative symptoms; such persons were thus considered as a “Non-Dissociative—Severe” (ND-S) class. Given identification of two dissociative classes, we found that requiring item scores of at least 3 (referring to “Quite a bit”) for at minimum one of the two depersonalization or derealization items rendered the optimal correspondence with placement in either of the two dissociative classes (sensitivity=77.60%, specificity=100%), giving a sample prevalence rate for the dissociative subtype of 25.49%. We refer to certain latent classes as having “moderate” symptom severity; however, this is only in comparison to other PTSD classes. In other words, all participants should be considered as having significant PTSD symptoms.

Comparison between the classes on the six latent factors identified by the principal axis factor analysis gave complementary results. In brief, group differences were observed in reference to latent factors 1–4. Specifically, the DISS-S and the ND-S classes obtained the highest *Reexperiencing* factor scores (factor 1); the EN-M and HYP-M classes obtained the highest and lowest *Emotional Numbing/Anhedonia* scores (latent factor 2), respectively; the DISS-M and DISS-S classes obtained the highest *Dissociation* factor scores (latent factor 3); and the EN-M and ND-S classes scored lowest on the *Negative Alterations in Cognition and Mood* factor (latent factor 4). No differences were observed in reference to predicted scores on the *Avoidance* or *Hyperarousal* factors; statistics are reported in Table 5.

**Table 5:** Differences between latent classes on latent factors identified by principal axis factor analysis

	a. Emotional numbing	b. Hyperarousal	c. Moderate D-PTSD	d. Severe D-PTSD	e. Severe ND-PTSD
1. Reexperiencing	−0.73 (0.41)^c^,^d^,^e^	−0.74 (0.38)^c^,^d^,^e^	−0.02 (0.52)^a^,^b^,^d^,^e^	1.83 (0.51)^a^,^b^,^c^,^e^	0.62 (0.46)^a^,^b^,^c^,^d^
2. Emotional numbing/Anhedonia	0.82 (0.60)^b^,^c^,^d^,^e^	−0.88 (0.66)^a^,^c^,^d^,^e^	−0.44 (0.72)^a^,^b^,^d^,^e^	0.01 (0.49)^a^,^b^,^c^	0.25 (0.63)^a^,^b^,^c^
3. Dissociation	−0.08 (0.70)^b^,^c^,^d^,^e^	−0.37 (0.71)^a^,^c^,^d^,^e^	0.91 (0.60)^a^,^b^,^d^,^e^	0.46 (0.46)^a^,^b^,^c^,^e^	−0.70 (0.62)^a^,^b^,^c^,^d^
4. Negative alterations in cognition and mood	−0.17 (0.77)^b^,^c^,^d^	0.20 (0.79)^a^,^e^	0.20 (0.81)^a^,^e^	0.18 (0.45)^a^,^e^	−0.26 (0.79)^b^,^c^,^d^
5. Avoidance	0.01 (0.91)	0.14 (0.71)	−0.29 (0.74)	−0.21 (0.59)	0.24 (0.73)
6. Hyperarousal	0.06 (0.82)	−0.25 (0.73)	0.30 (0.73)	0.09 (0.63)	−0.19 (0.78)

D=Dissociative, ND=Non-Dissociative

a Statistically significantly different from EN-M class

b Statistically significantly different from HYP-M class

c Statistically significantly different from DISS-M class

d Statistically significantly different from DISS-S class

e Statistically significantly different from ND-S class

All *p*'s<0.05 Bonferroni corrected.

### Differences between classes in other measures of dissociative experience

For differences between classes on the CDS, MDI, and MID, see Table 6. In brief, within Wave 1 participants, differences on the CDS were evident only in group comparisons involving the DISS-S class, who reported significantly greater *Anomalous Body Experience* and *Analomous Subjective Recall* in comparison with the three non-dissociative classes, significantly greater *Emotional Numbing* in comparison with the EN-M and ND-S classes, and significantly greater CDS *Alienation from Surroundings* in comparison with the HYP-M and ND-S classes.

**Table 6:** Differences between latent classes in other measures of dissociative experience

	a. Emotional numbing	b. Hyperarousal	c. Moderate D-PTSD	d. Severe D-PTSD	e. Severe ND-PTSD	*F*	*η* ^2^
CDS anomalous body experience (*n*=167)	2.27 (3.10)^d^	3.03 (4.21)^d^	5.74 (9.19)	9.04 (12.21)^a^,^b^,^e^	2.95 (5.42)^d^	4.69	0.10
CDS emotional numbing (*n*=167)	3.51 (3.69)^d^	3.50 (4.04)^d^	4.11 (5.71)	7.15 (7.05)^a^,^e^	3.09 (4.08)^d^	3.31	0.08
CDS anomalous subjective recall (*n*=167)	3.76 (3.06)^d^	3.50 (3.25)^d^	4.93 (4.77)	7.81 (6.54)^a^,^b^,^e^	3.81 (3.30)^d^	5.24	0.12
CDS alienation from surroundings (*n*=167)	3.46 (2.98)	2.63 (2.33)^d^	3.26 (3.80)	5.88 (5.12)^b^,^e^	3.28 (3.38)^d^	3.41	0.08
MDI depersonalization (*n*=82)	8.13 (3.95)^c^,^d^	9.72 (3.99)^d^	13.74 (5.58)^a^,^d^	20.38 (5.80)^a^,^b^,^c^,^e^	10.15 (4.79)^d^	12.17	0.39
MDI derealization (*n*=82)	10.88 (5.80)^d^	10.94 (3.92)^d^	14.47 (5.76)^d^	21.75 (5.70)^a^,^b^,^c^,^e^	11.76 (4.80)^d^	8.71	0.31
MDI emotional constriction (*n*=82)	11.67 (5.53)^d^	11.39 (4.43)^d^	14.47 (5.76)	19.50 (5.76)^a^,^b^	13.23 (6.07)	3.67	0.16
MDI disengagement (*n*=82)	14.62 (4.19)^d^	14.67 (4.61)	15.32 (6.06)	20.88 (5.74)^a^,^e^	12.85 (5.43)^d^	3.28	0.15
MDI memory disturbance (*n*=82)	9.83 (4.72)^d^	10.06 (4.57)^d^	14.00 (5.02)	17.50 (8.65)^a^,^b^,^e^	9.69 (4.70)^d^	5.05	0.21
MDI identity dissociation (*n*=82)	6.54 (3.66)^c^,^d^	7.50 (4.20)^c^,^d^	12.58 (6.12)^a^,^b^	16.75 (7.96)^a^,^b^,^e^	8.15 (4.77)^d^	8.71	0.31
MID depersonalization (*n*=169)	22.02 (16.63)^c^,^d^	26.94 (25.16)^c^,^d^	58.32 (22.30)^a^,^b^,^d^,^e^	83.33 (19.61)^a^,^b^,^c^,^e^	31.14 (24.24)^c^,^d^	34.29	0.46
MID derealization (*n*=169)	16.19 (14.84)^c^,^d^	22.67 (25.15)^c^,^d^	50.15 (19.40)^a^,^b^,^d^,^e^	67.80 (19.08)^a^,^b^,^c^,^d^	21.83 (23.40)^d^,^e^	30.72	0.43
MID trance (*n*=169)	23.44 (19.63)^c^,^d^	26.12 (24.26)^c^,^d^	50.37 (22.40)^a^,^b^,^e^	59.60 (30.71)^a^,^b^,^c^,^e^	32.00 (24.77)^c^,^d^	12.40	0.23
MID time loss (*n*=169)	5.60 (7.45)^c^,^d^	7.93 (7.65)^c^,^d^	14.66 (8.39)^a^,^b^,^e^	20.13 (12.69)^a^,^b^,^e^	8.89 (8.90)^c^,^d^	11.84	0.22

Class sizes per wave: EN-M (*n*_Wave__1_=41; *n*_Wave__2_=24; *n*_Wave__3_=48), HYP-M (*n*_Wave__1_=30; *n*_Wave__2_=18; *n*_Wave__3_=33), DISS-M (*n*_Wave__1_=27; *n*_Wave__2_=19; *n*_Wave__3_=38), DISS-S (*n*_Wave__1_=26; *n*_Wave__2_=8; *n*_Wave__3_=15), ND-S (*n*_Wave__1_=43; *n*_Wave__2_=13; *n*_Wave__3_=35). D=Dissociative, ND=Non-Dissociative

a Statistically significantly different from EN-M class

b Statistically significantly different from HYP-M class

c Statistically significantly different from DISS-M class

d Statistically significantly different from DISS-S class

e Statistically significantly different from ND-S class

CDS=Cambridge Depersonalization Scale, MDI=Multiscale Dissociation Inventory, MID=Multidimensional Inventory of Dissociation.

Within Wave 2, the DISS-M group reported higher MDI *Depersonalization* and *Derealization* symptoms than the EN-M group, whereas the DISS-S group scored higher than all four other groups. In comparison, referring to MDI *Disengagement*, the DISS-S group scored higher than the EN-M and ND-S classes. Referring to MDI *Emotional Constriction*, the DISS-S group ranked higher than the EN-M and HYP-M classes. Referring to MDI *Memory Disturbance*, the DISS-S group ranked higher than the EN-M, HYP-M, and ND-S classes. Finally, referring to MDI *Identity Dissociation*, the DISS-S class scored significantly higher than the three non-dissociative classes, whereas the DISS-M class scored higher than the EN-M and HYP-M classes.

Within Wave 3, MID *Depersonalization* and *Derealization* scores were significantly higher in both DISS classes as compared to the three non-dissociative classes, with the DISS-S and DISS-M groups further differing. In comparison, referring to MID *Time Loss* and *Trance*, whereas both dissociative classes again differed from the three non-dissociative classes, the DISS-S and DISS-M groups did not differ significantly from each other.

### Differences between classes in measures of non-dissociative symptoms

For differences between classes on the DERS, IIP, and MID-Emotional Suffering subscales, please see Table 7. Within Wave 1 participants, who were administered the DERS, no significant differences between groups were observed. Referring to Wave 2 participants who were administered the IIP, the only significant differences observed were for the DISS-S group to score higher in comparison with the EN-M and HYP-M groups. Finally, within Wave 3 participants, MID *Emotional Suffering* scores were significantly lower in the HYP-M group than all other groups, with no significant differences among the latter groups.

**Table 7:** Differences between latent classes in measures of presumed non-dissociative distress

	a. Emotional numbing	b. Hyperarousal	c. Moderate D-PTSD	d. Severe D-PTSD	e. Severe ND-PTSD	*F*	*η* ^2^
DERS (*n*=167)	88.37 (27.37)	85.73 (28.67)	87.70 (25.47)	88.33 (26.63)	99.81 (15.19)	1.16	0.03
IIP (*n*=82)	60.71 (22.20)^d^	55.61 (23.05)^d^	64.95 (27.68)	61.46 (13.76)^a^,^b^	91.13 (26.48)	3.50	0.15
MID-emotional suffering (*n*=169)	69.54 (25.78)^b^	50.64 (25.30)	67.55 (23.21)^b^	79.37 ( 21.43)^b^	87.47 (20.49)^b^	8.85	0.18

Class sizes per wave: EN-M (*n*_Wave__1_=41; *n*_Wave__2_=24; *n*_Wave__3_=48), HYP-M (*n*_Wave__1_=30; *n*_Wave__2_=18; *n*_Wave__3_=33), DISS-M (*n*_Wave__1_=27; *n*_Wave__2_=19; *n*_Wave__3_=38), DISS-S (*n*_Wave__1_=26; *n*_Wave__2_=8; *n*_Wave__3_=15), ND-S (*n*_Wave__1_=43; *n*_Wave__2_=13; *n*_Wave__3_=35). D=Dissociative, ND=Non-Dissociative.

a Statistically significantly different from EN-M class

b Statistically significantly different from HYP-M class

c Statistically significantly different from DISS-M class

d Statistically significantly different from DISS-S class

e Statistically significantly different from ND-S class

DERS=Difficulty in Emotion Regulation Scale, IIP=Inventory of Interpersonal Problems, MID=Multidimensional Inventory of Dissociation.

### Differences between classes in measures of childhood trauma exposure

For differences between classes on the CTQ, JVQ, and CTQ-S, please see Table 8. Within Wave 1 participants, there were no differences between classes for CTQ measures of emotional abuse and emotional neglect. However, the DISS-S group reported greater physical abuse and physical neglect histories than the EN-M group, and greater sexual abuse history than the HYP-M group as well as the ND-S group. In contrast, within Wave 2 participants, there were no differences between classes on the JVQ measures of any childhood trauma type. Finally, within Wave 3 participants, the DISS-S group reported greater physical and sexual abuse histories on the CTQ-S than all four other groups, excepting in the case comparing physical abuse history with the DISS-M group.

**Table 8:** Differences between latent classes in measures of childhood trauma history

	a. Emotional numbing	b. Hyperarousal	c. Moderate D-PTSD	d. Severe D-PTSD	e. Severe ND-PTSD	*F*	*η* ^2^
CTQ emotional neglect (*n*=167)	12.41 (5.18)	13.03 (4.92)	13.30 (5.52)	14.42 (6.05)	13.86 (5.72)	0.67	0.02
CTQ emotional abuse (*n*=167)	12.24 (6.22)	13.27 (5.98)	13.89 (5.91)	15.23 (7.10)	13.30 (6.49)	0.93	0.02
CTQ sexual abuse (*n*=167)	8.49 (5.75)	7.00 (4.68)^d^	10.44 (6.70)	11.84 (7.88)^b^,^e^	7.14 (4.97)^d^	3.78	0.09
CTQ physical abuse (*n*=167)	7.88 (4.26)^d^	7.80 (3.70)	9.96 (4.81)	11.54 (7.03)^a^	9.09 (5.31)	2.80	0.07
CTQ physical neglect (*n*=167)	7.59 (3.88)^d^	8.73 (3.36)	10.19 (4.18)	10.88 (5.60)^a^	8.79 (4.05)	2.91	0.07
JVQ conventional crime (*n*=82)	13.63 (10.15)	12.61 (8.84)	15.37 (11.00)	22.75 (12.91)	11.77 (7.42)	1.84	0.09
JVQ child maltreatment (*n*=82)	6.17 (5.88)	6.50 (5.16)	6.47 (6.14)	11.50 (7.29)	7.00 (4.38)	1.43	0.07
JVQ peer-sibling victimization (*n*=82)	9.17 (7.23)	11.17 (6.84)	9.32 (9.35)	17.75 (9.56)	8.92 (7.05)	2.12	0.10
JVQ sexual victimization (*n*=82)	7.67 (7.88)	7.06 (9.12)	8.05 (9.73)	10.63 (12.68)	7.85 (7.84)	0.22	0.01
JVQ witnessing violence (*n*=82)	7.54 (8.77)	10.11 (9.80)	10.11 (9.55)	15.88 (15.59)	8.00 (6.90)	1.21	0.06
CTQ-S emotional abuse (*n*=169)	3.40 (1.53)^d^	3.24 (1.54)^d^	3.63 (1.26)	4.67 (0.81)^a^,^b^	3.97 (1.38)	3.57	0.08
CTQ-S physical abuse (*n*=169)	1.96 (1.32)^d^	2.09 (1.37)^d^	2.82 (1.33)	4.00 (1.46)^a^,^b^,^e^	2.69 (1.53)^d^	7.52	0.16
CTQ-S sexual abuse (*n*=169)	2.42 (1.63)^d^	2.12 (1.29)^d^	2.45 (1.54)^d^	4.13 (1.41)^a^,^b^,^c^,^e^	2.77 (1.66)^d^	4.89	0.11

Class sizes per wave: EN-M (*n*_Wave__1_=41; *n*_Wave__2_=24; *n*_Wave__3_=48), HYP-M (*n*_Wave__1_=30; *n*_Wave__2_=18; *n*_Wave__3_=33), DISS-M (*n*_Wave__1_=27; *n*_Wave__2_=19; *n*_Wave__3_=38), DISS-S (*n*_Wave__1_=26; *n*_Wave__2_=8; *n*_Wave__3_=15), ND-S (*n*_Wave__1_=43; *n*_Wave__2_=13; *n*_Wave__3_=35). D=Dissociative, ND=Non-Dissociative.

a Statistically significantly different from EN-M class

b Statistically significantly different from HYP-M class

c Statistically significantly different from DISS-M class

d Statistically significantly different from DISS-S class

e Statistically significantly different from ND-S class

CTQ=Childhood Trauma Questionnaire, JVQ=Juvenile Victimization Questionnaire, CTQ-S=Childhood Trauma Questionnaire-Screen.

## Discussion

Within 557 persons self-reporting PTSD symptoms of at least moderate severity (PCL-5 scores ≥38) as assessed online, our LPA identified two latent classes comprising persons who endorse experiences of depersonalization and derealization of either moderate or high severity. The moderate and severe dissociative groups differed from three other latent classes of persons endorsing PTSD symptoms of moderate to high severity but who failed to endorse experiences of depersonalization and derealization. In addition, we found that persons who reported both PTSD and dissociative symptoms at high severity were also those who most often reported being physically and sexually abused as children. The results of the LPA were further supported by a principal axis factor analysis that identified a latent dissociation factor in addition to five other factors that parsed the symptomatology of DSM-5 PTSD in a manner generally consistent with that of the DSM-5 taxonomy and in agreement with the latent classes. Furthermore, correlations between the latent factors ranged from small to non-significant, indicating the importance of distinguishing between each symptomatic response to posttraumatic stress.

Collectively, our results provide continuing support for recognizing a subgroup of persons who experience depersonalization and/or derealization within the larger population of persons experiencing the signs and symptoms of DSM-5 PTSD. Our results suggest that among persons reporting at least moderate PTSD severity, yet surpassing the recommended clinical cut-off for the PCL-5, about one third (33%) endorse the presence of depersonalization and derealization, with 23% evidencing both moderate PTSD and dissociative symptoms overall, and the remaining 10% reporting both PTSD and dissociative symptoms of high severity. This differentiation between two subclasses of persons reporting moderate versus severe dissociative experiences is, to our knowledge, a novel finding of the present research. A cut-off score of three on either of the depersonalization or derealization items was also found to achieve optimal correspondence with placement in either of the dissociative classes, and would alternately suggest a sample prevalence rate of 26% for the dissociative subtype. The severe dissociative class (DISS-S) demonstrated very high difficulty with interpersonal relationships (i.e., high scores on the IIP) and emotion dysregulation (i.e., high scores on the DERS and MID Emotional Suffering) suggesting some resemblance to the notion of “Complex PTSD,” and arguments for recognizing the centrality of dissociation to complex presentations of PTSD have been put forth (e.g., Van der Hart, Nijenhuis, & Steele, 2005). A novel finding of the current research was that both dissociative PTSD classes evidenced greater severity of reckless/harmful behavior and difficulty remembering parts of a stressful experience. This finding was corroborated by our factor analysis, which demonstrated that these items load strongest on the *Dissociation* factor. Previous literature suggests the role of dissociation in both self-destructive behavior (Gratz, Conrad, & Roemer, 2002; Noll, Horowitz, Bonanno, Trickett, & Putnam, 2003; Van der Hart et al., 2005) and in experiences of amnesia for traumatic events (Wolf, Miller, et al., 2012, c.f. Wolf, Lunney, 2012). Future research should determine whether the presence of dissociative experiences is best conceptualized as a marker for more complex presentations of PTSD or is rather a marker for a more severe dissociative disorder.

Beyond our focus on dissociative symptomatology in PTSD, we also identified three non-dissociative latent classes composed of an overall severe PTSD class as well as two moderate PTSD classes, the first primarily characterized by frequent experiences of emotional numbing and anhedonia but low hyperarousal (specifically, hypervigilance and startle reactivity), termed an *Emotional Numbing* (EN-M) phenotype, and the second characterized by *low* emotional numbing and anhedonia but moderate hypervigilance and startle reactivity, termed a *Hyperarousal* (HYP-M) phenotype. The *EN-M* phenotype endorsed infrequent experiences of psychogenic amnesia, even though difficulty remembering parts of a stressful experience is considered a symptom of emotional numbing in DSM-5 PTSD. However, poor fit of psychogenic amnesia for the construct of emotional numbing is consistent with the original factor analytic research identifying a latent emotional numbing factor within PTSD symptomatology (King, Leskin, King, & Weathers, 1998), and with recent factor analytic work referring to the DSM-5 PTSD criteria (Armour et al., 2015; Gentes et al., 2014).

The HYP-M class also evidenced low levels of three DSM-5 Criterion E symptoms, considered to be part of DSM-5 *Hyperarousal*: “anger, irritability, or acting aggressively,” “risk taking behavior,” and “difficulty concentrating”; however, this class of individuals also evidenced frequent symptoms of reexperiencing, and also evidenced high levels of three other markers of hyperarousal (exaggerated startle response, hypervigilance, and insomnia). Such findings suggest that the latter three markers of hyperarousal may be more *specific* indicators of PTSD hyperarousal and may also be more strongly associated with intrusive recollections and strong anxiety responses to traumatic reminders. The replicability of these distinct non-dissociative PTSD presentations, as evidencing greater hyperarousal versus emotional numbing symptomatology, should be evaluated in clinical samples.

Results obtained from an exploratory factor analysis of PCL-5 items supported the recently proposed DSM-5 PTSD factor structure, with certain important caveats. *Reexperiencing* and *Avoidance* factors were identified which corresponded to the respective factors of the DSM-5 structure (Friedman, 2014). However, discrepancies from the DSM-5 structure were also observed. For one, the current study supported differentiating experiences of *Emotional Numbing and Anhedonia* from other *Negative Alterations in Cognitions and Mood*. Emotional numbing has been considered a crucial aspect of the reaction to overwhelming stress since the inception of PTSD in DSM-III (Brett, Spitzer, & Williams, 1998; Litz, 1992), and work by King and colleagues clearly demonstrated the importance of recognizing symptoms consistent with emotional numbing as distinct from general distress and effortful avoidance (King et al., 1998; Palmieri, Weathers, Difede, & King, 2007; Ruscio, Weathers, King, & King, 2002).

Another important outcome of our results was the support of the Simms’ *Hyperarousal* factor (Simms et al., 2002; Yufik & Simms, 2010), specifically, as consisting of two items assessing hypervigilance and exaggerated startle response, a simplified hyperarousal factor that therefore deviates significantly from the elaborated conceptualization of PTSD-associated hyperarousal as described in the DSM-5. Recognizing exaggerated startle and hypervigiliance as a specific factor may parse the heterogeneity of DSM-5 *Hyperarousal*, which contains many non-specific signs and symptoms of general distress (e.g., insomnia and concentration difficulties). The distinction of these two symptoms has received tentative support from recent theoretical and factor analytic work (Armour et al., 2015; Liu et al., 2014; Tsai et al., 2014). We hypothesize that at least the more interpersonal criteria of DSM-5 *Hyperarousal* (i.e., reckless/self-destructive behavior and, irritation, anger, or aggressive behavior) is more appropriately classified with the pervasive emotion dysregulation evidenced in *Negative Alterations in Cognition and Mood*, or as belonging to an *Externalizing Behavior* factor (Armour et al., 2015). Future research may consider using other statistical techniques, such as confirmatory factor analysis, to evaluate these hypotheses. In addition, future research may consider the clinical significance of alternate factor structures (as it pertains to the comorbidity between PTSD, affective and anxiety disorders, and dissociative disorders).

Although our results are generally consistent with our hypotheses, certain qualifications and study limitations should be acknowledged. Results concerning childhood trauma as a risk factor for the severe sub-class of the dissociative subtype were observed for the CTQ and CTQ-S but not for the JVQ. It is possible that the latter instrument may be insufficiently sensitive to the kinds of childhood trauma history most germane to the development of dissociative pathology; however, it is also likely that statistical power was insufficient to identify differences between classes. In addition, due to our lack of including a measure of trauma exposure other than those specific to childhood developmental trauma, we cannot conclude with certainty that our sample would meet diagnostic criteria for PTSD; in other words, high symptom levels may be present for certain participants even in the absence of trauma exposure. For example, rather than as PTSD classes per se, the *Emotional Numbing* class (EN-M) may be better conceptualized as a class primarily composed of depressed persons, while the *Hyperarousal* class (HYP-M) may be best conceptualized as an anxiety disorder class. Our research is also limited by the use of a cut-off score for PTSD that has been validated only in a military sample to date (Hoge et al., 2014). There is currently a lack of published research with the PCL-5, especially outside of military populations, and therefore no correspondingly reliable cut-off score for civilians has been developed, which is an urgent need in the literature.

Furthermore, as a general limitation, all of our measures were self-reports in nature; we recognize that stronger evidence will require multi-method and multi-informant approaches including via structured diagnostic interviews. In addition, although increasingly used in mental health research including in epidemiological studies of PTSD (e.g., Kilpatrick, 2013; Wolf et al., 2015), internet surveys may lack reliability relative to measures administered in the immediate presence of clinicians or researchers. Overall, the prevalence of PTSD in our original sample of 2507, as measured by the PCL-5, was high (i.e., 22%), which may partly owe to the self-report nature of assessment as well as recruitment via MTurk. Although clinical research in MTurk samples remains relatively sparse to date, one study found that prevalence of social anxiety disorder (SAD) was significantly higher in MTurk samples compared to the general population (Shapiro et al., 2013). Given that SAD and PTSD are frequently comorbid conditions (Collimore, Carelton, Hofmann, & Asmundson, 2010), it may be that rates of PTSD are also higher than normal in MTurk populations. Future research should consider administering measures to assess rates of comorbidity and implementing validity measures to remove participants who may be over-endorsing symptoms. Furthermore, our sample consisted primarily of females and Caucasians, which may limit the generalizability of our findings to male and non-Caucasian populations. Finally, our results require replication in research-diagnosed clinical samples before generalization to the latter groups is warranted.

It should be noted that the present study operationalized depersonalization primarily with respect to experiences of being outside one's body and/or experiences of bodily detachment, as has been similarly conducted in previous studies under DSM-IV. However, the diagnostic construct of depersonalization has itself undergone expansion within DSM-5, now referencing not only an altered sense of one's body but additionally alterations in a person's sense of time, experiences of emotional detachment/numbing, as well as alterations in self-perception more generally (Spiegel et al., 2011, 2013). Future research will need to use longitudinal designs to study the dissociative subtype, along with other dissociative disorders, across time, in order to determine whether depersonalization and derealization should continue to be considered the cardinal features of the disorder. Furthermore, future research may examine whether the dissociative subtype of PTSD, as compared to dissociative disorders, responds differently to various forms of treatment. It will also be important for psychobiological and psychophysiological studies to determine if there are specific markers for the dissociative subtype of PTSD versus dissociative disorders. Such research would prove vital to delineating a specific phenotype of dissociative PTSD.

We conclude that the present study provides further, tentative support for the presence of a dissociative subtype within the symptomatology of DSM-5 PTSD. Further research aiming to examine biomarkers associated with this subtype and to determine the clinical significance of this differential diagnosis for the treatment of PTSD and dissociative pathology is highly recommended.

## Footnotes

1 The current research is part of a larger web survey project investigating the relationship between childhood trauma exposure, PTSD symptoms, and dissociation from the perspective of a new theoretical model termed the “4-D model” (Frewen & Lanius, 2015). Toward that broader goal, included within the web survey were measures germane to the trauma and dissociation fields including posttraumatic symptoms, interpersonal and emotion difficulties, childhood traumatic experiences, and trait dissociation. Analyses directly pertinent to examination of the “4-D model,” however, will be reported elsewhere.
